# Laser Powder Bed Fusion and Hot Forging of 316L Stainless Steel: A Hybrid Additive Manufacturing Approach for Enhanced Performance

**DOI:** 10.3390/ma18214909

**Published:** 2025-10-27

**Authors:** Sambhaji Kusekar, James Elder, Jay Desai, Showmik Ahsan, Daniel Young, Ganesh Walunj, Tushar Borkar

**Affiliations:** 1 Department of Mechanical Engineering, Cleveland State University, Cleveland, OH 44115, USA; s.kusekar@vikes.csuohio.edu (S.K.); j.a.elder50@vikes.csuohio.edu (J.E.);; 2 Department of Mechanical and Materials Engineering, Wright State University, Dayton, OH 45435, USA; ahsan.3@wright.edu (S.A.); daniel.young@wright.edu (D.Y.); 3 Engineering Technology Department, Buffalo State University, Buffalo, NY 14222, USA

**Keywords:** 316L stainless steel, laser powder bed fusion, forging, hybrid additive manufacturing, mechanical properties, microstructure

## Abstract

Forging plays a crucial role in various industries, including aerospace, automotive, oil and gas, and defense. We investigated the effect of post-processing forging on microstructural and mechanical properties of 316L stainless steel forging preforms fabricated by laser powder bed fusion. The as-built samples were subjected to hot forging in order to refine the microstructure and enhance mechanical performance. Detailed characterization was performed using Electron Backscatter Diffraction, Scanning Electron Microscopy, Energy Dispersive Spectroscopy, Tensile testing, and Hardness Testing. Substantial grain refinement (up to 97%) was observed, in addition to a reduction in porosity. The forging process effectively transformed the columnar grain morphology into equiaxed grains, increased yield and ultimate tensile strengths of 560 MPa and 740 MPa, representing 27% and 32% improvements, respectively, with a corresponding decrease in elongation to 32% from 47%. The horizontally built samples achieved the highest yield strength of 605 MPa but slightly lower UTS 710 MPa, representing 32% and 5% increment and decrease in ductility to 28% from 37.5%. These trends reflect the combined effects of work hardening and grain refinement, which enhance strength at the expense of ductility.

## 1. Introduction

Hybrid manufacturing is an integrated approach that utilizes new additive manufacturing (AM) methods to enhance and synergize with conventional manufacturing technologies. Traditional manufacturing is typically optimized for large-scale production and is built on decades of knowledge, technology, and a deep understanding of specific materials’ processing–property relationships. This enables a high degree of manufacturing reliability and material performance, particularly in the area of forged metal components. However, traditional manufacturing is less optimal for producing small numbers of components or when rapid fabrication or design iterations are required. Hybrid manufacturing addresses these concerns by incorporating AM into the traditional manufacturing flow. Rather than using AM as a standalone manufacturing method, hybrid manufacturing leverages the capabilities of AM to alleviate the bottlenecks inherent in conventional processes. This can provide synergistic effects where the design flexibility and manufacturing capabilities of AM expand the capabilities of traditional manufacturing while maintaining its advantages, reducing lead time, energy consumption, tooling costs, and material waste while maintaining the materials’ performance and reliability of conventional methods.

Many high-performance stainless steel 316L components are produced by traditional forging. 316L is a low-carbon version of the 316 austenitic stainless steel, with a maximum carbon level of 0.03%. It contains chromium (16–18%), nickel (10–14%), and molybdenum (2–3%) as major alloying elements with minor additions of manganese, silicon, nitrogen, phosphorus, and sulfur. Chromium enhances corrosion resistance, improves mechanical properties, and supports high-temperature performance. Molybdenum increases corrosion resistance, especially against pitting and crevice corrosion. Nickel enhances the cold deformation capacity. Even at cryogenic temperatures, nickel stabilizes the austenitic microstructure, thereby increasing the steel’s ductility and toughness [1,2]. A low-carbon composition is typically used to prevent chromium carbide formation, thereby improving corrosion resistance and reducing the risk of hot cracking. It also enhances ductility and toughness, making the fabricated components more reliable for demanding structural applications. This composition is well-suited for applications that require superior corrosion resistance, high durability, and strength. High formability allows for relatively easy manufacturing compared to many other high-alloy metals. High strain hardening rates and resistance to cracking allow for the development of high-strength properties through plastic deformation. The energy, automotive, defense, aerospace, chemical, transportation, and medical sectors commonly employ 316L, which is used to fabricate a wide variety of components such as airplane engine parts, landing gear, missile casings, structural vehicle parts, armor plating, naval hardware, and pressure vessels [3,4,5]. The traditional route for the 316L manufacturing of large high-performance components is casting, followed by hot forging and subsequent post-processing. Forging steps are required since the yield strength of annealed 316L is typically in the range of 150 to 300 MPa, which prevents its use for load-bearing or structural applications [6,7].

AM of metal components typically yields anisotropic grain sizes and higher defect densities compared to cast/annealed microstructures. While there is potential to utilize metal AM components directly in demanding applications, recent AM studies show challenges in obtaining uniform material properties due to the presence of anisotropy, porosity, columnar grain structures, and layered structure defects in AM materials. Therefore, additional subsequent processes are required to achieve optimal mechanical properties. Brennan et al. [8] demonstrated that the microstructural deficiencies present in fusion-based and sintering-based AM material can be counteracted by defect-mitigation methods such as hot isostatic pressing (HIP). Jiang et al. [9] demonstrated that AM parts have a coarse-grained microstructure and/or a large number of micro voids. Gordon et al. [10] studied AM-induced defects such as keyholes, lack of fusion defects, balling, and solidification porosity. Lewandowski et al. [11] studied the low-fatigue performance of AM-processed materials in comparison to wrought and cast materials, which was attributed to as-deposited surface roughness and process-induced flaws. Kok et al. [12] elucidated the driving factors that result in material anisotropy and microstructure heterogeneity. Luecke et al. [13] demonstrated how processing parameters, such as horizontal and vertical build direction, affect mechanical behavior under vertical tension or compression loading. Yusuf et al. [14] revealed that the layer-by-layer nature of AM processes creates non-uniformly distributed porosity, and that crack propagation and fatigue resistance were strongly affected by void distribution. Dzugan et al. [15] used miniature samples of various AM-processed alloys to document properties that rely on both orientation and location [16].

The published literature suggests other ways to address materials anisotropy in metallic AM materials. Cold rolling, hot compression, and hot forging can all be used to increase the strength of 316L stainless steel by creating substantial homogeneous plastic deformation, resulting in dense networks of dislocations. High-temperature processes can directly encourage recrystallization, or cold processing can be followed by annealing to induce grain refinement. As described in the Hall–Petch relation, smaller grain sizes typically result in higher yield strength, although this is accompanied by a corresponding reduction in ductility, leading to the well-known strength–ductility trade-off [6,17,18,19].

While forging is typically utilized to produce many of the highest-performance, mission-critical metal components in the aerospace and defense industries, practical technological, and logistical hurdles create lengthy procurement times. Lead times have doubled or even tripled in specific material categories across the Defense Industrial Base (DIB). Much of this delay is due to the expense and complexity of multi-stage forging processes. Additionally, component expense is increased by the need to fabricate components in an oversized state and utilize machining to obtain the final geometry, which results in waste of material. New technologies such as AM, coupled with forging operations, could support more resource-efficient production and supplement traditional process chains. According to existing studies [20,21], fabrication of a Ti-6Al-4V external landing gear assembly using AM resulted in a material weight saving of up to 16.7% per part for the exterior landing gear system.

A combined manufacturing route that utilizes AM metal preform fabrication, followed by forging operations, represents a promising hybrid manufacturing approach. Metal AM can be used to rapidly fabricate near-net-shape preforms, eliminating the need for iterative forging steps or die creation, thereby accelerating the manufacturing process [9]. Not only can this reduce tooling requirements, but it could also significantly reduce material waste. The final hot forging step can create a high-performance component, densifying and refining the microstructure, correcting any remaining flaws, and meeting precise dimensional specifications. Thus, this synergistic approach can address the lead time and production cost problems inherent in traditional 316L stainless steel forgings [22,23,24,25]. The directional grain flow and porosity reduction inherent in the final forging step can facilitate the production of finished components that meet or exceed design criteria for strength, ductility, and fatigue life [26,27]. This form of hybrid manufacturing is a focus of current research interest. AM technology has advanced rapidly to meet the growing demand for intricate and high-performance parts, particularly in the aerospace sector. Alcoa Corporation has developed a novel hybrid process called Ampliforge^TM^, which combines conventional forging with AM [28,29]. When compared to products created solely through AM, the process significantly reduces the amount of material input, shortens lead time, and enhances the quality of 3D-printed parts.

Despite significant advances in hybrid AM techniques, a detailed understanding of how AM-induced anisotropy, porosity, and grain morphology evolve during the subsequent forging of AM 316L stainless steel remains limited. Existing studies primarily address either AM processing or post-processing individually, without systematically correlating AM for low-volume forged applications, microstructural evolution, and resulting mechanical performance after forging. Therefore, there is a critical need to establish the relationship between AM build characteristics and their transformation after forging to optimize preform design for hybrid additive manufacturing applications.

In this study, we investigated the critical parameters in a hybrid AM/forging manufacturing process for 316L stainless steel alloy. We utilized laser powder bed fusion (L-PBF) and a drop forging finishing process. We observe the effects of L-PBF fabrication parameters on the resulting mixed equiaxed and columnar grains in the scanning direction, and columnar grains in the build direction. The resulting anisotropic AM metal microstructures were then subjected to the hot forging process, followed by characterization of density, hardness, and tensile strength. Optical microscopy (OM), scanning electron microscopy (SEM), energy dispersive spectroscopy (EDS), electron beam backscatter diffraction (EBSD), and x-ray diffraction (XRD) were utilized to comprehensively characterize the defect and microstructural evolution during this hybrid manufacturing process.

## 2. Materials and Methods

### 2.1. Sample Preparation

Gas-atomized SS316L stainless steel powder with a particle size ranging from 15 to 53 µm was purchased from Carpenter Additive LLC (Tanner, Alabama, USA). Table 1 shows the chemical composition provided by the supplier.

**Figure 1:**
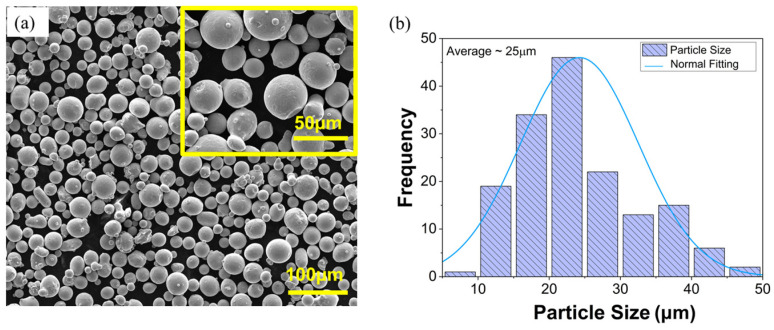
(**a**) SEM morphology of 316L stainless steel powder with magnified image in inset and (**b**) particle size distribution of 316L stainless steel powder.

**Figure 2:**
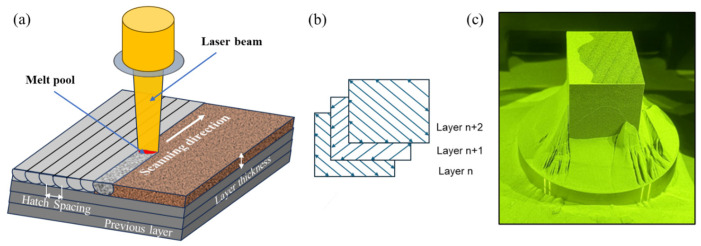
Schematic showing (**a**) the laser powder bed fusion L-PBF process, (**b**) the L-PBF stripe scanning strategy, and (**c**) the final L-PBF part.

Powder morphology was characterized by scanning electron microscopy (SEM, Inspect F50, Thermo Fisher Scientific, Waltham, MA, USA) operating at 20 kV. The particle size distribution was determined using ImageJ software, as shown in Figure 1. L-PBF samples were additively manufactured using a TRUMF-TruePrint 1000 3D Laser Metal Fusion system Plymouth Township, MI 48170, USA with parameters, as shown in Table 2. The printing process employed a stripe scanning strategy in which the laser trajectory was rotated 51° between consecutive layers. The sample geometry was a 60 mm × 60 mm × 80 mm cuboid. Figure 2 illustrates a schematic of the printing process, scanning strategy, and the geometry of the as-built part. Two L-PBF blocks were fabricated. One was used in an as-built state while the other was subjected to a forging process.

### 2.2. Hot Forging Process

**Figure 3:**
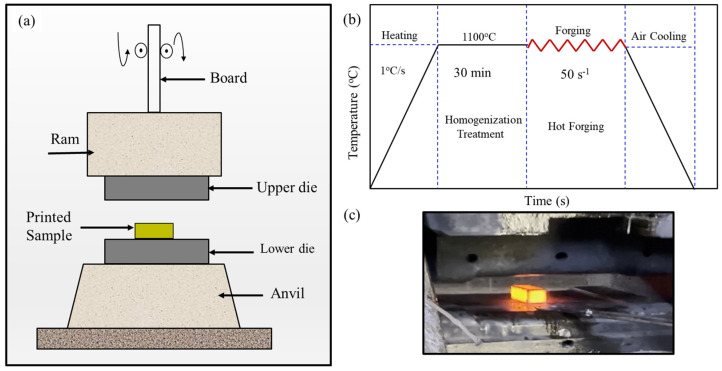
Schematic showing (**a**) a schematic of the hammer hot forging process, (**b**) the utilized thermal cycle including homogenization treatment, and (**c**) an image of the forging process.

One L-PBF as-built sample was flat-die hot-forged using a 14k hammer forging at Canton Drop Forge (Canton, OH, USA). A schematic of the hot forging process is shown in Figure 3a. Before hot forging, the sample was homogenized by solution annealing heat treatment for 30 min at 1100 °C (heating rate 1 °C/s). The purpose of this heat treatment was to induce a single-phase austenitic crystal structure and prevent the formation of chromium carbide, which can cause unwanted corrosion sensitization. Afterwards, the solution heat-treated sample was subjected to multi-directional hammer forging and then air cooled, as shown in Figure 3b,c. The process involved hammer forging without reheating steps. Two faces were hammer forged with three passes on each face.

### 2.3. Microstructure Characterization

Small specimens (12 mm × 12 mm × 5 mm) were cut out by wire electric discharge machining (EDM), obtained from Mitsubishi MC Machinery Systems Inc. (Elk Grove Village, IL, USA), along the build direction and scan direction of the as-built samples and as-forged samples. Then, samples were mounted using graphite conductive mount media and prepared using silicon carbide (SIC) grinding paper (Buehler, IL, USA) (120, 240, 400, 600, 800, and 1200 grit). Final polishing was performed using colloidal silica (0.04 μm) for 15 min, followed by ultrasonic cleaning for 10 min. X-ray diffraction (XRD) analysis was carried out using a Rigaku Ultima III diffractometer (Irvine, CA, USA) (Cu-Kα, 40 kV, 1°/min, 0.02° scan step). Microstructural examinations and elemental analysis were performed using a scanning electron microscope (SEM, FEI Inspect F50, Waltham, Massachusetts, USA) equipped with an energy-dispersive x-ray spectroscopy (EDS) system from Oxford Instruments (Model: X-MAX). EBSD analysis was performed on an SEM/EBSD system (JEOL JSM-7900F, EDAX Hikari+). The grain size distribution from FE-SEM micrographs was quantified using ImageJ software, with measurements performed on approximately 50 grains for each sample.

### 2.4. Mechanical Properties Testing

Sample densities were measured using the Archimedes method with deionized (DI) water as a working medium. The relative density was computed for each sample based on the ratio of the measured bulk density to the theoretical density (7.98 g/cm^3^). The Archimedes density measurement was performed in accordance with ASTM B962-17 standards. To minimize surface tension effects and bubble entrapment, the samples were ultrasonically cleaned for 15 min, dried using compressed air, and subsequently measured in deionized water. The suspended and immersed masses were recorded using a Sartorius analytical balance (Manufacturer: Sartorius; Model: QUINTIX64-1S; Serial No.: 0037750155; Version BAC: 00-50-02.02 CN: 1701; Version APC: 01-70-03.03 CN: 9352) integrated with an Archimedes density determination kit (Bohemia, NY, USA). The relative density was calculated as 99.91 ± 0.06%, where the uncertainty represents the 95% confidence interval based on three repeated measurements. A Wilson VH1202 microhardness tester (Buehler, IL, USA) was used to measure Vickers hardness at predetermined intervals from the surface. The distance between indentations was maintained at 2.50D (diagonal). The hardness measurements were performed at a load of (HV0.5) with dwell time of 15 s. The profile path and indentation positions are illustrated schematically in Figure 15, and error bars representing the standard deviation have been added. A total of 10 readings (n = 10) were taken for each condition to ensure statistical reliability. As-built and as-forged materials were machined into cylindrical dogbone specimens (ASTM E8 standard, gauge length 36 mm, diameter 9 mm). These samples were extracted in both the build and scanning directions. A Tinius Olsen 60K Super L Universal Testing Machine was utilized for tensile testing under position rate control conditions (0.05″/min through yield, 0.50″/min through fracture).

## 3. Results

### 3.1. Microstructure Characterization

#### 3.1.1. Optical Porosity Analysis

**Figure 4:**
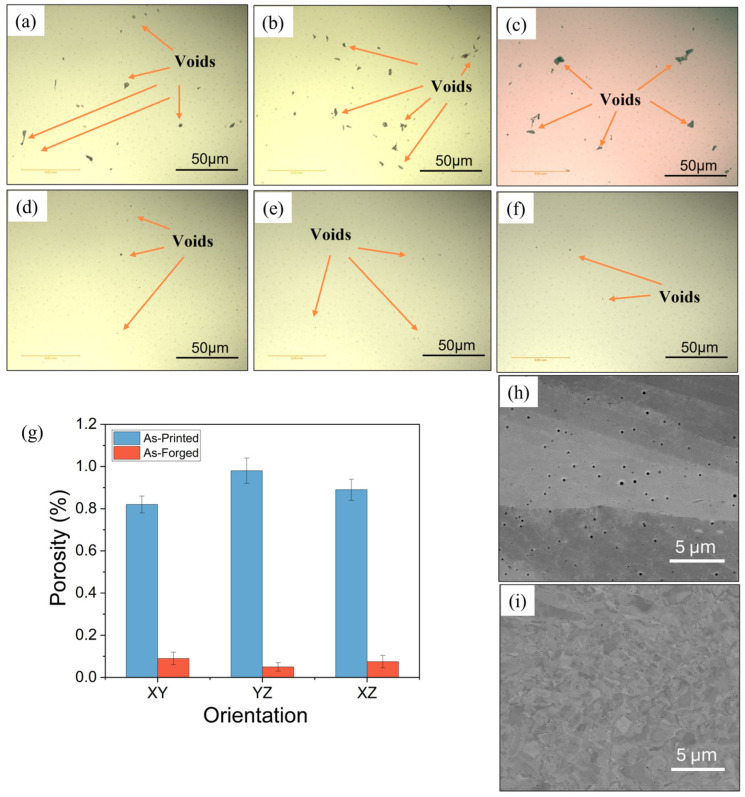
Optical images of porosity variation for L-PBF 316L: L-PBF as-built in the (**a**) XY plane (scanning direction), (**b**) YZ plane (build direction), (**c**) XZ plane (build direction), (**d**) XY plane (scanning direction), (**e**) YZ plane (build direction), (**f**) XZ plane (build direction), (**g**) quantitative porosity analysis for all studied samples, and (**h**) SEM micrograph showing pore morphology in forging direction (**h**) as-built (**i**) forged.

Low-magnification (50X) optical micrographs are shown in Figure 4a–f, providing a clear overview of the porosity distribution in as-built and hot-forged samples. Porosity is calculated according to the technique implemented by Cai et al. [30], based on the black regions identified in the optical microscopy (OM) (VH1202, Buehler, Lake Bluff, IL, USA) image using ImageJ. OM images were analyzed covering an area of approximately 0.0356 mm^2^, as indicated by the scale bar. The pore size distribution ranged between 1 and 8.5 µm, with an average circularity of 0.46 µm, indicating slightly elongated voids typical of gas entrapment in AM materials. We acknowledge that OM provides only a 2D surface assessment and does not capture subsurface or interconnected porosity. This is the limitation of this 2D technique. The estimated percentage porosity for as-built and hot-forged samples is shown in Figure 4g. The presence and quantity of porosity have been shown to significantly reduce tensile and fatigue strength in AM materials. Joshi et al. [31] studied defect structure, such as lack of fusion porosity, in L-PBF Ti-Al-4V and observed that strength and strain to failure decreased with increasing void defect presence. The size, shape, and proximity of the pores to the surface also have a significant effect on the severity of stress concentration. Higher stress concentrations are produced by larger pores and those nearer to the surface, reducing fatigue life and accelerating crack initiation [32,33,34]. Furthermore, porosity reduces the effective load-bearing cross-section [35]. The hot forging process, however, effectively reduces the size and population of voids and can lead to significant improvement [9]. In L-PBF as-built samples studied in the build direction (YZ plane), more porosity was observed in comparison to the scanning/transverse direction (XY plane). This is due to the layered nature of L-PBF material, where each layer is added and fused to the previous one, which can result in incomplete fusion between layers. Additionally, thermal gradients, high cooling rates, and uneven heat distribution can lead to keyhole porosity or gas entrapment, particularly in the direction perpendicular to the build direction. Thus, comprehensive microstructural analyses have demonstrated that the lack of fusion pores, one of the most common and harmful forms of porosity in L-PBF, typically localizes along layer borders and is oriented perpendicular to the build direction [35].

Hot forging involves heating the AM samples above recrystallization temperatures and compressing them to the desired shape under high pressure. This results in a significant reduction in porosity, as shown in Figure 4d–f. In addition, SEM micrographs of as-built and forged conditions with pore morphology are shown in Figure 4h–i. Thus, hot forging is likely an effective technique to consolidate AM components. The synergy between recrystallization and high-pressure deformation facilitates the healing of internal defects, eliminates porosity, and produces a denser and more homogeneous microstructure. Thus, it is possible that hot forging could eliminate the anisotropic effects caused by the uneven distribution of pores during the AM process. For these materials, in the scanning direction (XY) and build direction (YZ), the mean porosity area fraction was significantly reduced by 94% and 89%, respectively, after hot forging.

#### 3.1.2. SEM Analysis and EBSD Grain Orientation Analysis

**Figure 5:**
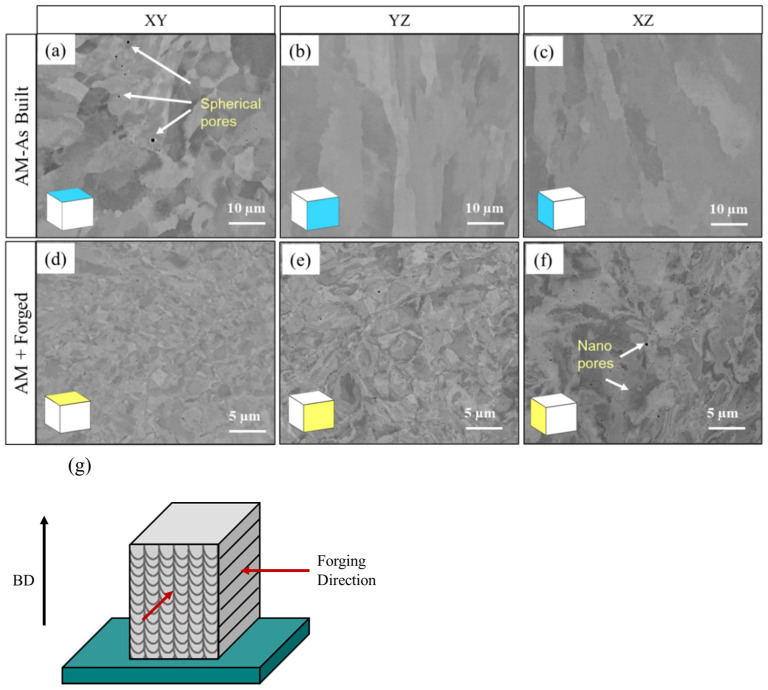
SEM–BSED image of L-PBF as-built (**a**–**c**) and L-PBF + forged samples (**d**–**f**) in the XY, YZ and XZ planes, respectively, and (**g**) hammer forging direction.

**Figure 6:**
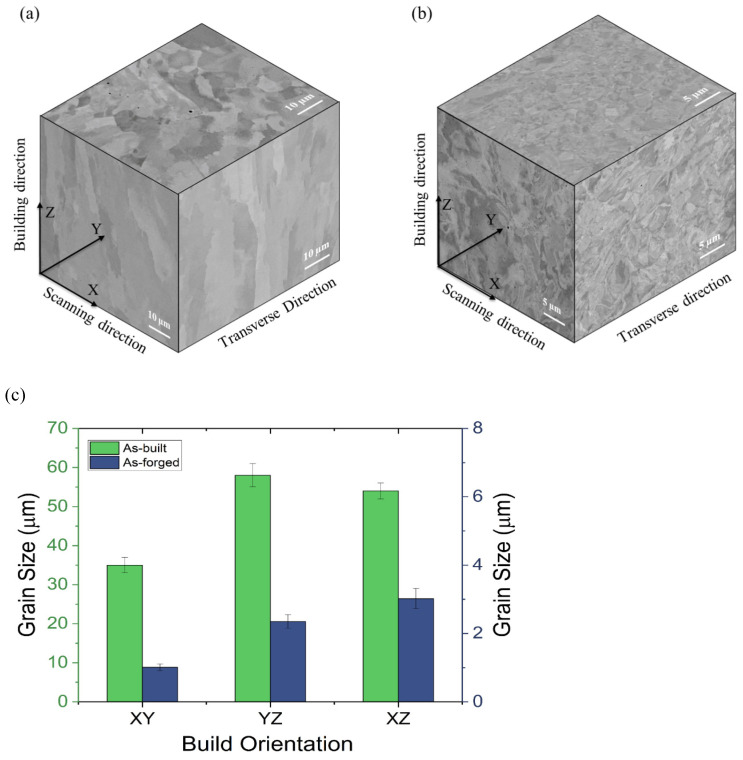
(**a**) A 3D representation of the as-built microstructure, (**b**) 3D representation of the forged microstructure, and (**c**) comparison of average grain size between as-built and forged samples.

A detailed microstructure analysis was performed on as-built and forged samples by FESEM. The SEM images are shown in Figure 5a–f. Grain size analysis was performed on all the samples in all three planes (XY, YZ, and XZ) using ImageJ software. The average grain size measurements for the microstructures shown in Figure 6a,b are presented in Figure 6c. In addition maximum grain size and average grain size are given in Table 3. The samples exhibited a mix of columnar and equiaxed grains in the XY plane (in the scanning direction) with an average grain size of 35 µm, and columnar grains in the YZ and XZ planes (in the build direction), with average grain sizes of 58 µm and 54 µm, respectively. Directional heat flow, epitaxial growth, and fast solidification conditions that inhibit equiaxed nucleation are considered to be the causes of columnar grain structures in L-PBF 316L. Additionally, higher laser powers can cause more melting and resolidification, leading to longer grains [36].

A significant grain refinement (94 to 97% reduction in grain size) is observed in all directions after hot forging, indicating effective recrystallization due to plastic deformation. In the forging direction (XY), shown in Figure 5g, the most refined grain structure was observed with an average grain size of 1.01 µm, whereas the YZ and XZ vertical planes show average grain sizes of 2.35 µm and 3.02 µm, respectively. Maximum grain size values exhibited similar effects, with a reduction from 43.25 µm to 4.42 µm in the XY plane and from 63.52 µm to 7.20 µm in the YZ plane. These results clearly demonstrate that the forging process improves grain uniformity and significantly reduces grain size, in addition to removing much of the microstructural anisotropy induced by AM. The finer and more equiaxed grain morphology achieved through forging is expected to translate into improved mechanical properties, such as increased fracture toughness and fatigue strength, aligning with the objectives of preform optimization for industrial forging routes.

Scanning electron microscopy, energy dispersive spectroscopy, and electron beam backscatter diffraction measurements were performed using a JEOL 7900FLV scanning electron microscope and an EDAX EDS/EBSD (Hikari+) system. Specimen surfaces were metallographically prepared using conventional grinding and polishing methods, followed by vibratory final polishing. EBSD images were obtained at 100× magnification with a 1 micron step size and Kikuchi images were obtained at a rate of approximately 150 fps. Image processing was performed using EDAX (V4.6)/OIM (V8.1.0) software. Energy dispersive spectroscopy was utilized to study the chemical homogeneity of the as-built and forged material. Representative elemental data are shown in Figures 12 and 13.

**Figure 7:**
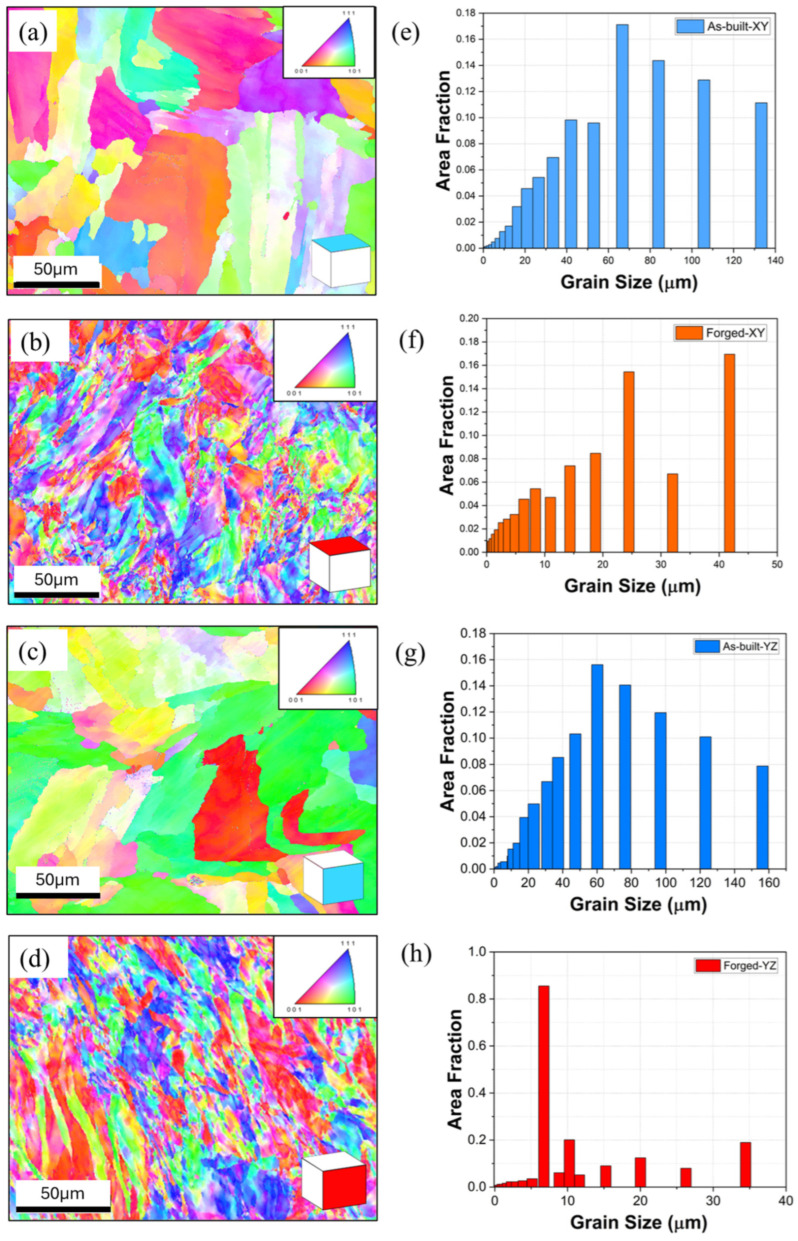
EBSD IPF maps and grain size distributions of L-PBF processed 316L stainless steel in XY and YZ planes: (**a**) as-built XY, (**b**) forged XY, (**c**) as-built YZ, and (**d**) forged YZ; (**e**) grain size: as-built XY, (**f**) forged XY, (**g**) as-built YZ, and (**h**) forged YZ.

In order to observe the effect of forging on the as-built microstructure, electron backscatter diffraction (EBSD) imaging was performed on the XY (build direction surface) and YZ planes for both as-built and forged materials, as shown in Figure 7. Figure 7a–d shows the microstructures for as-built XY, forged XY, as-built YZ, and forged YZ, respectively. It should be noted that the build direction for Figure 7b,d is oriented towards the top of the image. Grain size analysis data are presented in Figure 7e–h, The as-built XY microstructure exhibits a relatively coarser, large columnar grained microstructure. Little in-plane texturing is evident which is consistent with layer-by-layer rotation of laser scan direction.

After forging, the resulting microstructure (Figure 7b,d) exhibits significant grain refinement driven by dynamic recrystallization (DRX). In the direction of forging, approximately 85% of the grains were refined below 6.5 µm shown in Figure 7d,h, attributed to the higher local strain and accumulated plastic deformation along the primary forging axis. This is a promising outcome from the AM preform design perspective, as it enables the evaluation of strain distribution within the forged structure to achieve a tailored grain structure through controlled preform geometry and deformation conditions. Statistical fluctuation is evident in the grain size histograms, likely due to the number of quantified grains. While large grain size data sets would resolve this issue, the trends in these data are clear. Additionally, spatial inhomogeneity was also clearly observed. This may be due to non-uniform strain, thermal gradients, and varying local deformation during the forging process. This likely explains the discrepancy between calculated grain size from SEM imaging and EBSD imaging. The low-magnification EBSD images in Figure 7 clearly show bands of high grain refinement interspersed with regions of relatively unrefined microstructure, while Figure 5 shows SEM images of microstructure from recrystallized regions, with correspondingly smaller calculated grain sizes.

**Figure 8:**
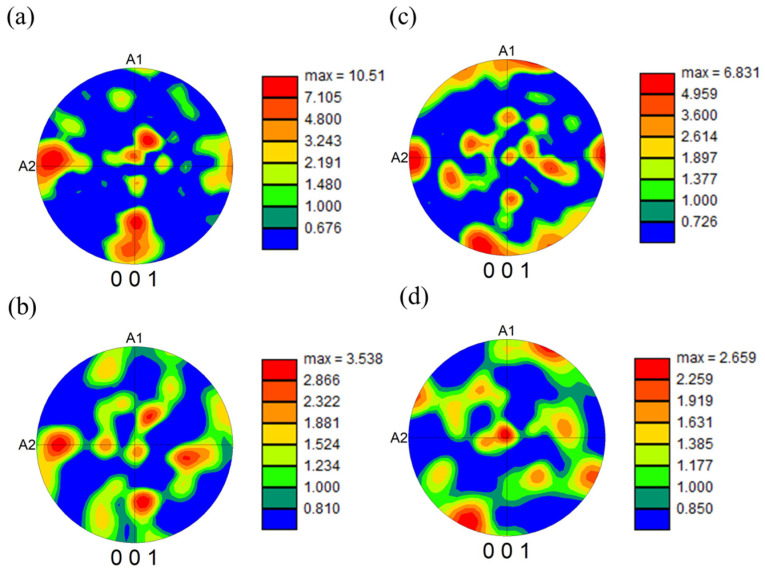
Pole figures of (**a**) XY as-built, (**b**) XY forged, (**c**) YZ as-built, and (**d**) YZ forged microstructures.

The as-built microstructure exhibits relatively low crystallographic rotation within each grain, consistent with the presence of low amounts of residual stresses and plastic deformation. In contrast, after forging, significantly more plastic deformation and residual stresses are evident as forging processes impose significant macroscopic strains causing increased dislocation density and residual stresses. In the YZ plane, the as-built microstructure still exhibits a relatively large grain structure, but some texturing in the Z-direction is evident, which is consistent with the high thermal gradients present in the build direction during laser powder bed fusion. After forging, significant grain refinement is evident; however, some regions with texturing in the Z-direction remain visible. More grain refinement was observed in the XY plane as opposed to the YZ plane. Figure 8 shows pole figures for these EBSD data sets. A relatively small amount of (001) texturing is evident for both as-built and forged samples.

The persistence of texture along the YZ plane and partial recrystallization are indeed attributed to insufficient strain accumulation and localized strain heterogeneity during 3 pass hammer forging. It can be systematically investigated through mitigation strategies such as (i) employing multi-pass hammer forging with controlled intermediate reheating to promote uniform strain distribution, (ii) optimizing deformation temperature and strain rate to enhance dynamic recrystallization, and (iii) implementing post-forging solution and annealing treatments to achieve complete texture randomization [37,38,39,40].

**Figure 9:**
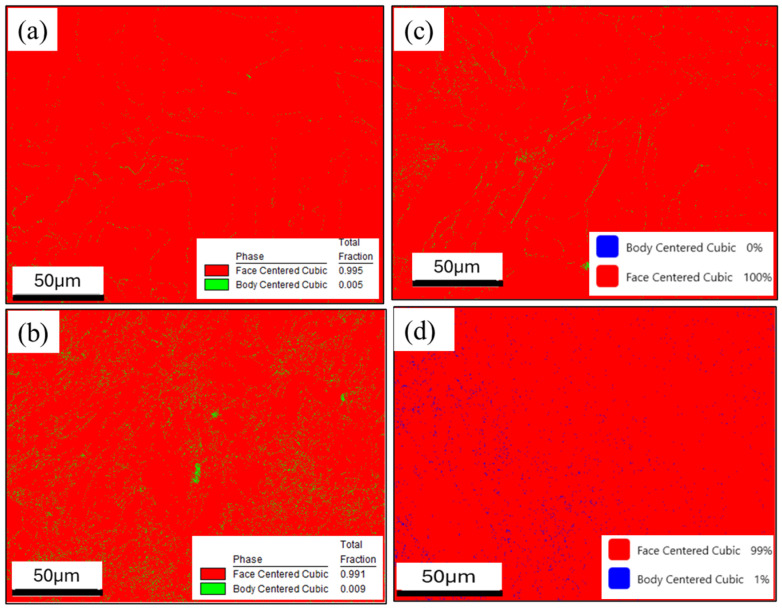
EBSD phase maps of polished and unetched L-PBF (**a**) as-built XY, (**b**) forged XY, (**c**) as-built YZ, and (**d**) forged YZ microstructures.

Figure 9 shows the phase maps for the same samples, suggesting the presence of approximately 4% δ-ferrite formed at cell/dendrite boundaries within an FCC (austenite) matrix, for the as-built and forged XY planes, and for the as-built YZ plane. The forged YZ plane exhibited less detected ferrite at under 1%. These images suggest that δ-ferrite may be localized near high-angle grain boundaries, as these high-energy regions favor the segregation of alloying elements and the formation of secondary phases. If so, this residual phase could have deleterious impact on the toughness and corrosion resistance of the material. However, the level of detected ferrite in these images is not supported by the x-ray diffraction data, which are more consistent with an entirely austenitic structure, especially since 1% ferrite should be above the XRD detection limit. These phase maps could be partially explained by EBSD image analysis artifacts. During phase identification by TEAM/OIM software, data pixels at grain boundaries or other microstructural defects can be misidentified as other crystal structures. Thus, the XRD results likely impose a very low upper limit on the residual ferrite present in these materials.

**Figure 10:**
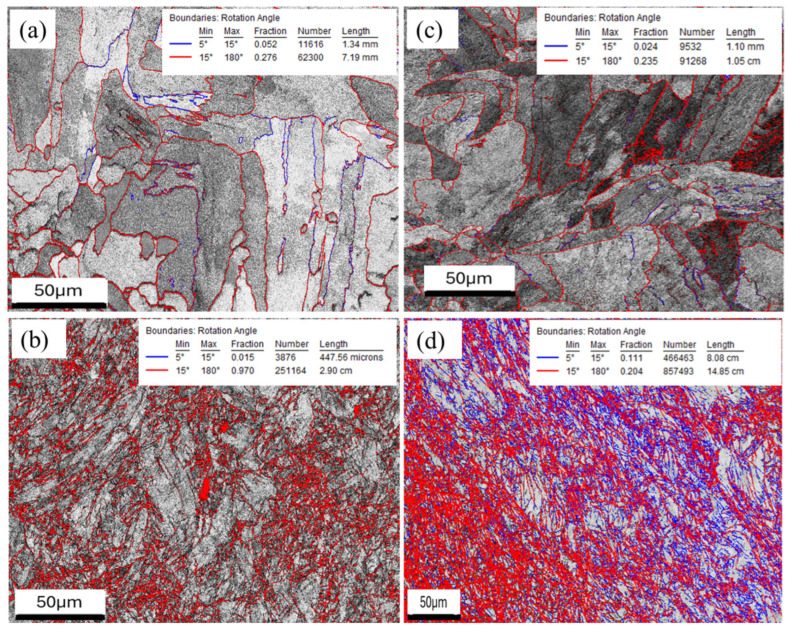
(**a**) EBSD characterization of polished and unetched (**a**) as-built XY, (**b**) forged XY, (**c**) as-printed YZ, and (**d**) forged YZ microstructures.

Figure 10a–d shows the computed grain boundary maps for as-built XY, forged XY, as-built YZ, and forged YZ microstructures, respectively. OIM software was used to calculate grain boundary angle populations between 5° and 15° and 15° and 180°. Figure 9a shows a microstructure with relatively clean high-angle grain boundaries with a small population of medium-angle grain boundaries, consistent with a large-grained, equiaxed microstructure with a low degree of plastic deformation. After forging, Figure 10b shows a significant increase in the high-angle grain boundary length, while the medium-angle grain boundary length is still low.

A more pronounced increase in grain boundary length can be observed in Figure 10c,d, with discrete high-angle grain boundaries and a sparse network of medium-angle grain boundaries in the as-built state and major increases in both medium and high-angle grain boundaries in the forged state. Medium-angle grain boundaries were defined as boundaries with a lattice misorientation in the range of 5–15°, while 15–180° degrees was defined as the range for large-angle grain boundaries.

For this sample, it is possible that specimen preparation played a role in the marked increase in medium-angle grain boundaries; however, it is also possible that this image displays the presence of bands of regions with high amounts of plastic deformation. This latter explanation is supported by a comparison of the Figure 7d and Figure 10d, since the regions of high medium-grain boundary density spatially match with the presence of relatively larger grains that likely exhibited less recrystallization during forging. This would be consistent with the presence of higher levels of un-recrystallized lattice defects, leading to the OIM algorithm identifying higher concentrations of medium-angle grain boundaries.

Figure 7, Figure 8 and Figure 10 provide insights into the local deformation behavior that occurred during compression. Figure 7b shows local regions of recrystallized grains after forging in the XY plane, which appear to be relatively evenly distributed in orientation, while Figure 10d shows the recrystallization behavior in the YZ plane. In the YZ plane, the recrystallized material appears to manifest as bands oriented approximately 45° to the forging direction. This could be consistent with deformation banding effects that occur during hot compression. While discernable, this effect is relatively subtle and is not strongly evident in the pole figures shown in Figure 8. However, the grain boundary maps in Figure 10 show a clear pattern with relatively even distribution of both medium-angle and high-angle grain boundaries in the XY plane, in contrast to the significant increase in medium-angle grain boundaries in the YZ plane, in the forged material, oriented at approximately 45° to the compression axis.

**Figure 11:**
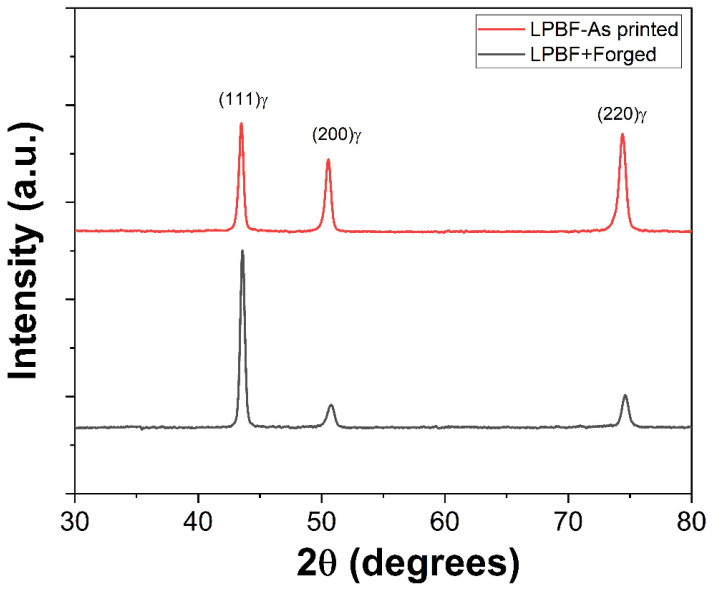
XRD patterns of L-PBF as-built and forged samples.

Figure 11 shows XRD patterns of L-PBF as processed and forged samples, which indicate that the L-PBF as-built 316L stainless steel retains high residual stress and a fine microstructure due to rapid cooling, as evidenced by the broader diffraction peaks as compared to forged material. The L-PBF processed samples only displayed the (111), (200), and (220) peaks of single-phase austenite (FCC), which is consistent with the reported literature [3,41]. Post-forging leads to peak sharpening, suggesting defect reduction, stress relief, and reduced micro strain/defect density. Notably, the material maintains strong dominant peaks of face-centered cubic (FCC) γ-Fe (austenite) phase stability after forging, which is beneficial for structural integrity in 316L applications.

**Figure 12:**
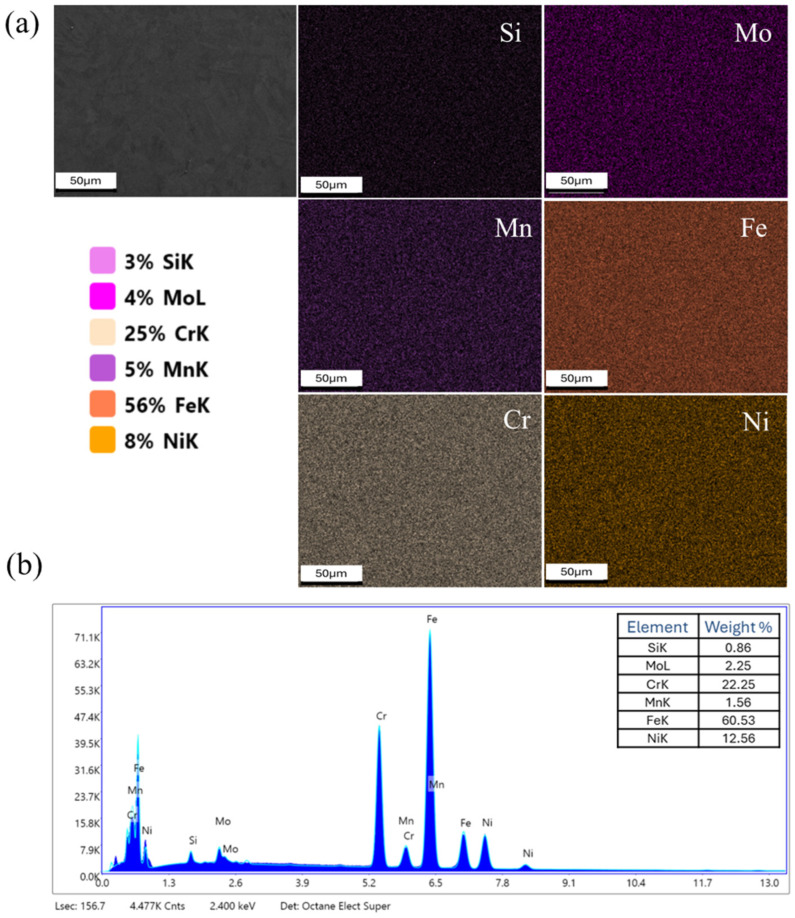
(**a**) EDS elemental mapping of the L-PBF as-built sample and (**b**) EDS spot analysis spectrum.

Elemental characterization was performed for the primary alloying elements: Fe, Cr, Ni, Mn, Mo, and Si. Other elements with lower content or lighter atomic weights were excluded. Figure 12a shows a representative spatial elemental map set for the as-built material. While there is no evidence of significant second-phase formation, it is worth noting that the EDS technique, at this imaging length scale, cannot resolve submicron phases, either at grain boundaries or within grain interiors. However, these qualitative maps indicate a high degree of chemical homogeneity at the microstructural level. Spot EDS analysis of the as-built material is shown in Figure 12b, indicating compositional ranges consistent with 316L stainless steel. While this method has an accuracy range of 2–5 atomic percent and thus provides only qualitative compositional information, the results are consistent with 316L material fabricated by laser powder bed fusion, which typically maintains consistent composition before and after processing.

**Figure 13:**
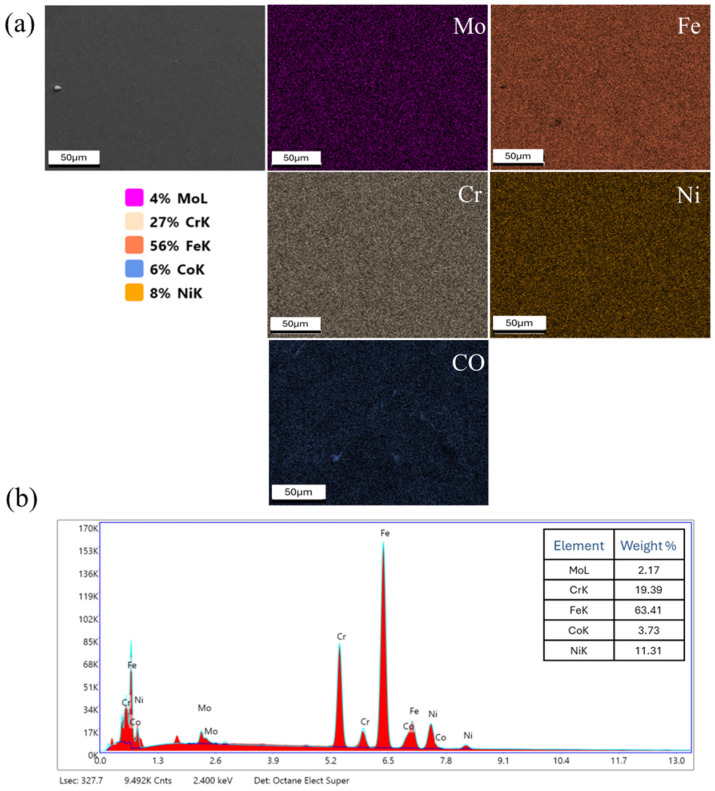
(**a**) EDS elemental mapping of the L-PBF + forged sample and (**b**) EDS spot analysis spectrum.

Figure 13a,b shows the elemental maps and spot EDS data after forging, with similar results. These data suggest that the forging process did not result in any microstructural-level precipitation of second phases at the grain boundaries, which would be consistent with a relatively short dwell at high temperatures.

### 3.2. Mechanical Properties and Fractography

#### 3.2.1. Microhardness

**Figure 14:**
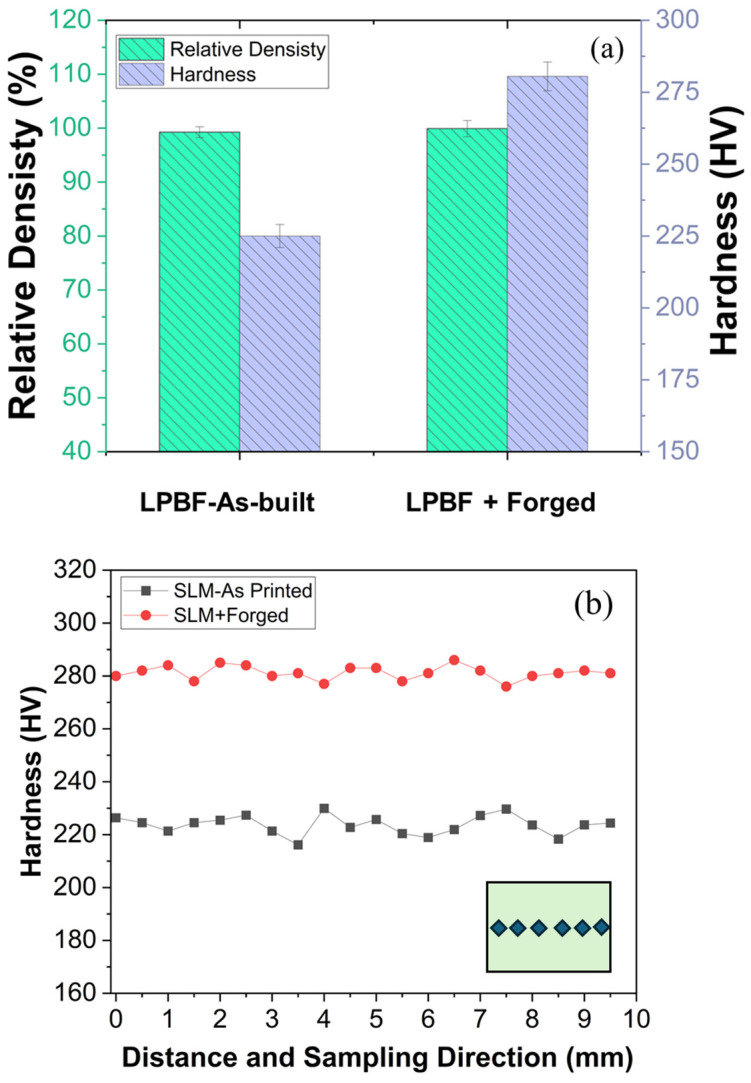
(**a**) Density and hardness comparison and (**b**) hardness profile comparison of L-PBF as-built sample and forged material.

The density and hardness measurements for as-built and forged materials are shown in Figure 14a. The initial high density achieved in the as-built state (99.15%) is characteristic of optimized L-PBF parameters, which promote effective powder melting and minimal porosity, correlating directly with a resultant hardness of 225 HV. However, residual micro voids and partially bonded regions may persist within the microstructure after the L-PBF process, which are not entirely eliminated even with optimized scanning strategies and energy input [42].

**Figure 15:**
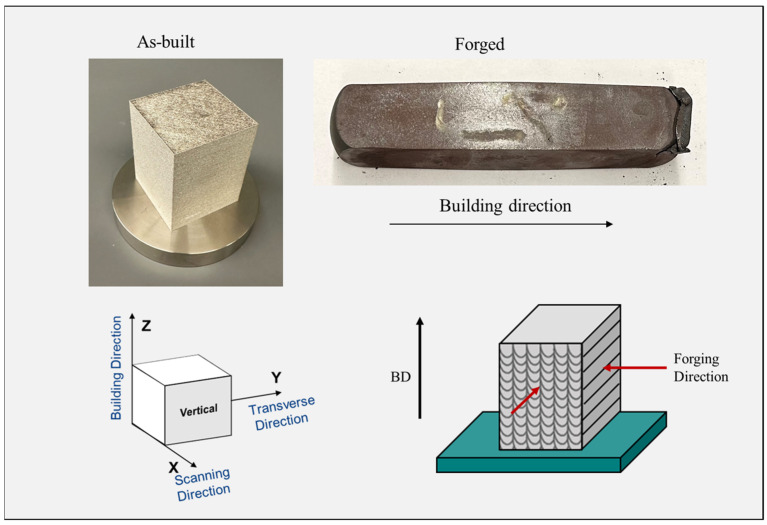
Actual blocks of 316L stainless steel alloy in the L-PBF as-built and hot-forged conditions with build direction and forging direction schematics.

The forging process serves to densify and work-harden the material, reducing residual porosity and increasing the relative density to 99.91%. A significant deformation of 161% along the Z-direction and a true strain of 96% were observed, as shown in Table 4 and in Figure 15. Post-forging, this promoted grain boundary movement, pore closure, and the potential for recrystallization, all of which likely contributed to the substantial increase in hardness to 280.4 HV. Furthermore, to compare the variation in hardness between the as-built and forged samples, a hardness profile was obtained and is shown in Figure 14b. The as-built sample exhibited slight variation in hardness, whereas the forged sample showed a more uniform hardness distribution, indicating improved consistency after forging.

#### 3.2.2. Tensile Properties

**Figure 16:**
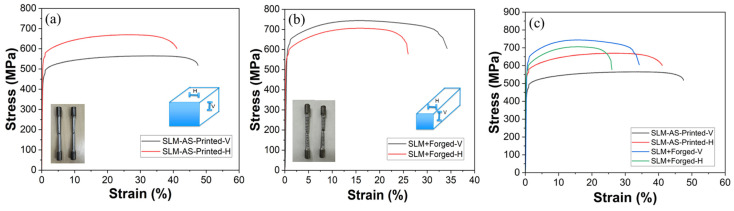
Room-temperature tensile deformation behavior of AM 316L stainless steel samples at various conditions: (**a**) as-built in the XY (horizontal) and Z (vertical) directions, (**b**) hot-forged in the XY (horizontal) and Z (vertical) directions, and (**c**) comparison of as-built and forged properties in the horizontal and vertical directions.

A room temperature true stress–strain curve for various conditions is shown in Figure 16. The tensile properties of 316L stainless steel processed by L-PBF in both the as-built and forged conditions are shown in Table 5 for both vertical (V) and horizontal (H) build orientations. Compared to annealed wrought 316L stainless steel samples, the as-printed L-PBF samples showed higher YS and UTS in both orientations. This can be attributed to their cellular dendritic substructure and inherent residual stresses. It has been hypothesized that the layered nature of the L-PBF process induces defects along planes perpendicular to the vertical build direction, leading to higher internal stress concentration and cross-section reduction during tension in the build direction. This can ultimately lead to lower yield strength and ultimate tensile strength values [43,44] for vertically aligned L-PBF samples. The comparison of AM + forged 316L stainless steel with wrought annealed and wrought + forged is also shown in Table 5.

After forging, the data showed an increase in vertical YS to 560 MPa and UTS to 740 MPa, representing 27% and 32% increases, respectively. However, this strength improvement came at the expense of ductility, with elongation dropping to 32% (15% reduction). This trend aligns with conventional strengthening mechanisms such as work hardening and grain refinement, which typically reduce ductility. The horizontal as-built samples demonstrated higher yield and tensile strength compared to their vertical counterparts: 550 MPa and 677.5 MPa, respectively, although there was also a slight reduction in elongation (37.5%).

In hot forging or hot deformation of AM 316L stainless steel, the competition among DRX-driven softening, pore closure (densification) hardening, and texture evolution ultimately shapes the strain hardening trajectory. As forging proceeds, DRX nucleation and growth reduce dislocation density and tend to promote ductility, but that benefit can be offset if a significant fraction of the work is consumed in closing pores and generating stresses around defect sites, especially when DRX is spatially non-uniform; concurrently, texture inherited (or partially retained) from the deformation path biases slip system activation and strain partitioning, affecting the slopes and transitions among classical hardening stages I–III, which must have retained work hardening and dropped ductility. In Stage I—texture and orientation dominate initial dislocation multiplication before DRX becomes active; in Stage II—recovery, substructure evolution, and the onset of DRX nuclei compete with pore closure stresses and hardening in defective regions, which can reduce the dσ/dε slope; in Stage III—as DRX proceeds toward saturation and densification nears completion, the microstructure coarsens or stabilizes under refined, more uniformly recrystallized grains and more randomized texture, and the flow stress approaches a steady state.

**Figure 17:**
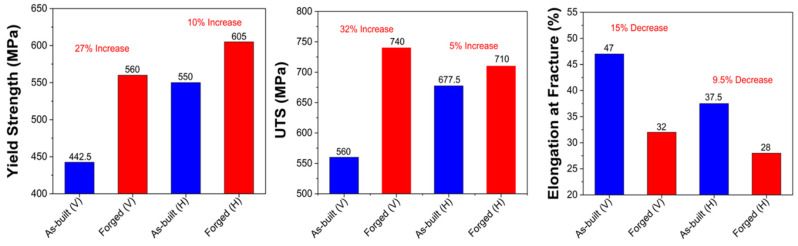
Percentage increase in YS and UTS and decrease in elongation after forging 316L stainless steel.

Following forging, horizontally built samples achieved the highest yield strength among all conditions at 605 MPa, though the UTS were slightly less than the vertically built samples at 710 MPa and 740 MPa, respectively. The elongation further decreased to 28%, the lowest among all tested conditions. This reduction in ductility, combined with elevated strength, suggests that forging homogenizes and densifies the microstructure. The percentage increase in YS and UTS after forging, for both vertical and horizontal builds, is shown in Figure 17. Compared to wrought annealed 316L stainless steel (206 MPa YS, 500 MPa UTS, and 40% elongation), all L-PBF processed conditions, both as-printed and forged, demonstrated significantly higher strength values.

#### 3.2.3. Strain Hardening Behavior

**Figure 18:**
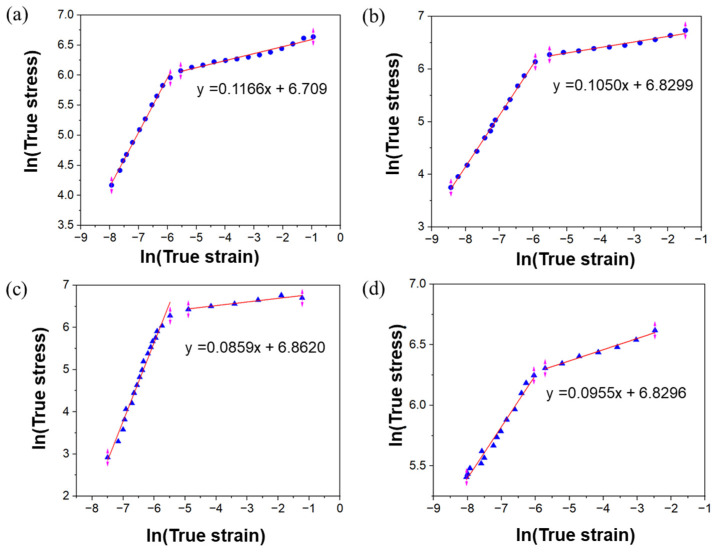
ln (true stress) vs. ln (true strain) plot of 316L stainless steel (**a**) as-printed vertical direction, (**b**) as-printed horizontal direction, (**c**) forged vertical direction, and (**d**) forged horizontal direction.

The Hollomon equation is used to describe the strain hardening behavior of materials during plastic deformation in tensile testing, as shown in Equation (1). It relates true stress and true plastic strain in the plastic region (after the yield point) [47,49]:

(1) $$\sigma =k\varepsilon_{p}^{n}$$

where σ is true stress (MPa), k is a strength coefficient (MPa), and ε_p_ is true plastic strain. n is the strain hardening exponent. The Holomon equation is used to fit the true stress–strain data, as shown in Figure 18a–d. All samples showed a two-stage strain hardening behavior, meaning the relationship between lnσ and ln ε was nonlinear, with a transition strain between deformation stages. To ensure physical consistency, the Hollomon equation was fitted exclusively to the true plastic regime (0.0025 ≤ ε_p_ ≤ 0.37) after removing the elastic strain component (ε_p_ = ε − σ/E). The strain hardening exponent and strength coefficient are obtained from plastic region fitting and are shown in Table 6. Similar work hardening is reported in the literature [47,50,51]. The L-PBF + forged sample in the vertical and horizontal direction showed higher strain hardening compared to wrought 316L, likely due to a higher degree of grain structure refinement and increased dislocation density from the hybrid forging process.

#### 3.2.4. SEM Fractography Analysis

**Figure 19:**
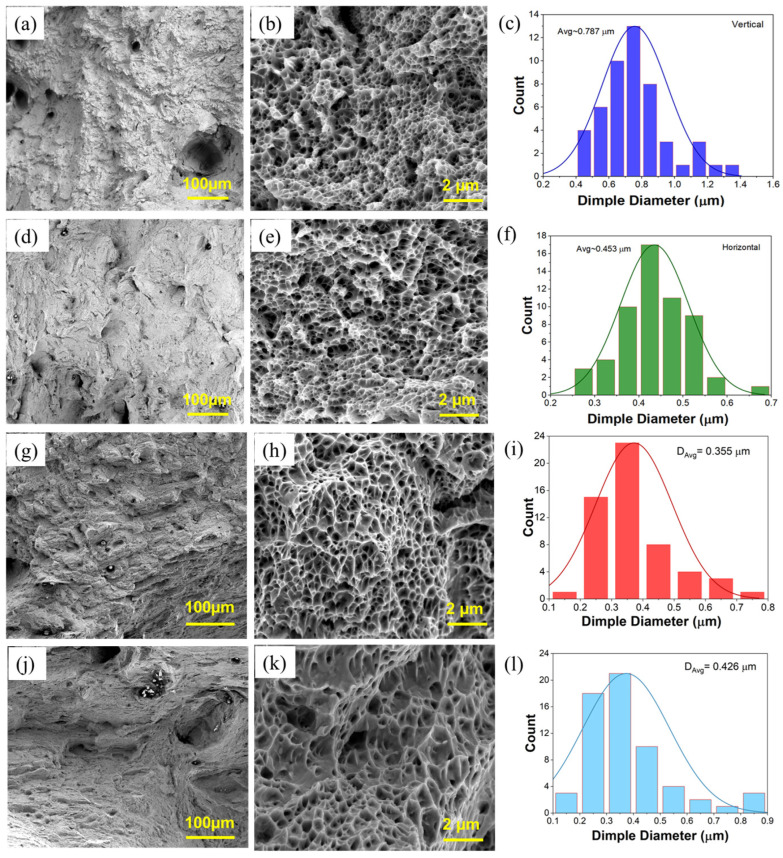
SEM fracture images and dimple size distribution of L-PBF as-built 316L in (**a**–**c**) build direction, (**d**–**f**) scanning direction (perpendicular to build), and L-PBF + forged in (**g**–**i**) build direction and (**j**–**l**) scanning direction (perpendicular to build).

Fractography analysis was conducted to analyze the fracture surfaces of the tensile-tested specimens of as-built and forged samples. SEM–BSED micrographs of fractured specimens for as-built and forged samples are presented in Figure 19a,b,d,e, corresponding to samples from the vertical and horizontal orientations. The presence of dimple surfaces indicates a ductile fracture mechanism. Dimples result from the nucleation, growth, and coalescence of micro voids during plastic deformation. Small dimples (< 0.5 µm in diameter) are observed due to the refined cellular substructure in L-PBF processed 316L alloys [46,52]. In addition, large crater-like voids and porosities were observed in as-built fractured specimens.

The variation in dimple size across build orientations for as-built and forged samples was quantified and is illustrated in Figure 19c,f for as-built samples and Figure 19i,l for forged samples extracted from the vertical and horizontal orientations.

A distinct trend is observed between build orientation and post-processing in the average dimple diameters obtained from fracture surfaces. The as-built vertical build samples had a greater average dimple diameter of 0.787 µm, whereas the as-built horizontal samples had an average of 0.453 µm. The forged samples had averages of 0.355 μm for the horizontal orientation and 0.426 μm for the vertical orientation, showing a notable refinement in dimple size by 55% and 6%, respectively.

After forging, the dimple morphology was refined, indicating a shift toward a finer and more uniform microstructure, which is typically associated with altered mechanical properties. In particular, a smaller dimple size typically represents a higher YS and UTS, as well as a lower elongation at fracture, which indicates a decrease in ductility. This inverse relationship between dimple size and strength properties suggests that the material is both efficiently densified and microstructurally homogenized during the forging process, resulting in increased strength at the expense of some ductility.

## 4. Conclusions

A combination of AM and hot forging represents a promising hybrid manufacturing approach for low-volume forging applications. We have investigated the effect of hot forging on AM-processed 316L stainless steel preforms to assess the feasibility of integrating AM into conventional forging routes, which could lead to reduced manufacturing times, material waste, energy consumption, and tooling costs.

Hot forging reduced porosity in L-PBF processed 316L stainless steel samples by 94% (XY) and 89% (YZ), effectively mitigating anisotropic pore distributions. Additionally, it enhanced hardness from 225 HV to 280.4 HV, producing a more uniform hardness profile indicative of effective densification, pore elimination, and microstructural refinement.

SEM and EBSD imaging revealed significant grain refinement (85% grains were observed below 6.5 µm in the YZ plane) after the forging process. In the as-built condition, the XY plane exhibited large equiaxed grains, whereas the YZ plane showed directionally oriented grains. Following forging, both planes developed refined microstructures, with grain refinement being more pronounced in the XY plane.

EDS analysis confirmed chemical homogeneity in both as-built and forged 316L stainless steel, with no significant formation of second phases. Minimal (001) crystallographic texturing was present in both as-built and forged conditions. However, the as-built YZ plane exhibited mild Z-direction texturing due to thermal gradients during the L-PBF process. Post-forging, some textural features remained, especially in the YZ plane, indicating incomplete recrystallization in certain regions.

Analysis of grain boundary misorientation distributions revealed an increase in high-angle grain boundaries (HAGBs) post-forging, consistent with recrystallization and plastic deformation. The forged YZ plane exhibited a notable increase in medium-angle grain boundaries (MAGBs), which may be attributed to regions of retained deformation and incomplete recrystallization.

After forging, the vertically built samples exhibited increased yield and ultimate tensile strengths of 560 MPa and 740 MPa, representing 27% and 32% improvements, respectively, with a corresponding decrease in elongation to 32% from 47%. The horizontally built samples achieved the highest yield strength of 605 MPa but slightly lower UTS 710 MPa, representing 32% and 5% increments and a decrease in ductility to 28% from 37.5%. These trends reflect the combined effects of work hardening and grain refinement, which enhance strength at the expense of ductility.

The L-PBF forged sample in the vertical and horizontal direction showed higher strain hardening compared to wrought 316L due to refined grain structure and increased dislocation density from the forging process. Fractography revealed refined dimple morphology after forging, indicating improved microstructural uniformity and densification, which contributed to increased strength and reduced elongation.

## Figures and Tables

**Table 1:** Chemical composition of the 316L stainless steel powder (wt.%).

Element	Fe	Cr	Ni	Mo	Mn	Si	N	O	P	C	S
wt.%	Balance	16–18	10–14	2–3	2	1	0.1	0.1	0.045	0.03	0.03

**Table 2:** Process parameters by the equipment manufacturer for 316L stainless steel.

Laser Power (W)	Scanning Speed (mm/s)	Hatch Spacing (µm)	Layer Thickness (µm)	Laser Spot Size (µm)	Atmosphere
180	350	125	40	55	Argon

**Table 3:** Average and maximum grain sizes of as-built and as-forged samples (Sampling size n= 50).

Specimens	Plane/Direction	Average Grain Size (µm)	Maximum Grain Size (µm)
As-built	XY-Horizontal	35	43.25
	YZ-Vertical	58	63.52
	XZ-Vertical	54	59.22
As forged	XY-Horizontal	1.01	4.42
	YZ-Vertical	2.35	7.20
	XZ-Vertical	3.02	10.37

**Table 4:** Dimensions of L-PBF as-built sample and forged sample.

SLM	X (Length) (mm)	Y (Width) (mm)	Z (Height) (mm)	Increase in Z After Forging	True Strain ln(L/Lo)
As-built	60	60	80	-	-
Forged	42	48	209	161%	0.96

**Table 5:** Tensile properties comparison of as-built, forged, and wrought 316L stainless steel samples.

Sample	YS (MPa)	UTS (MPa)	Elongation (%)	Reference
L-PBF-As-built (V)	442.5	560	47	Present work
L-PBF-As-built (V)	400	520	20	[45]
L-PBF + Forged (V)	560	740	32	Present work
L-PBF-As-built (H)	550	677.5	37.5	Present work
L-PBF-As-built (H)	380	500		[45]
L-PBF + Forged (H)	605	710	28	Present work
L-PBF + Forged (V)	380	500	15	[39]
L-PBF + Forged (H)	345	530	17	[45]
L-PBF + Forged (V)	351	540	18.3	[45]
Wrought	261.1	562.2	62.8	[46]
Wrought-Annealed	260	581	59.2	[47]
Wrought + Forged (3 Pass)	450	896	26	[38]
Wrought + Forged (9 Pass)	600	1060	8.0	[38]
Hot Isostatic Pressing (HIP)	331	656	43	[48]

**Table 6:** Hollomon equation parameters for L-PBF as-built, L-PBF + forged, and wrought conditions.

Sample	Orientation	k (MPa)	95% CI (k)	n	95% CI (n)	R^2^	Reference
L-PBF	V	812.40	780–862	0.1166	0.1166 ± 0.010	0.986	Present study
	H	915.98	880–973	0.1050	0.1050 ± 0.010	0.995	
L-PBF+ Forging	V	953.36	909–004	0.0859	0.0859 ± 0.010	0.989	Present study
	H	915.98	880–972	0.0925	0.0925 ± 0.010	0.994	
Wrought	T_d_	1178.5	-	0.348	-	0.996	[47]
	R_d_	1195.1	-	0.349	-	0.994	

## Data Availability

The original contributions presented in this study are included in the article. Further inquiries can be directed to the corresponding authors.
